# Fluorine-Functionalized Polyphosphazene Immunoadjuvant: Synthesis, Solution Behavior and In Vivo Potency

**DOI:** 10.3390/molecules28104218

**Published:** 2023-05-21

**Authors:** Harichandra D. Tagad, Alexander Marin, Ruixue Wang, Abdul S. Yunus, Thomas R. Fuerst, Alexander K. Andrianov

**Affiliations:** 1 Institute for Bioscience and Biotechnology Research, University of Maryland, Rockville, MD 20850, USA; 2 Department of Cell Biology and Molecular Genetics, University of Maryland, College Park, MD 20742, USA

**Keywords:** vaccine adjuvants, polyphosphazenes, fluorine-containing pharmaceuticals, protein-polymer interactions, supramolecular self-assembly, hepatitis C virus, hydrolytic degradation, polyelectrolytes

## Abstract

The inclusion of fluorine motifs in drugs and drug delivery systems is an established tool for modulating their biological potency. Fluorination can improve drug specificity or boost the vehicle’s ability to cross cellular membranes. However, the approach has yet to be applied to vaccine adjuvants. Herein, the synthesis of fluorinated bioisostere of a clinical stage immunoadjuvant—poly[di(carboxylatophenoxy)phosphazene], PCPP—is reported. The structure of water-soluble fluoropolymer—PCPP-F, which contains two fluorine atoms per repeat unit—was confirmed using ^1^H, ^31^P and ^19^F NMR, and its molecular mass and molecular dimensions were determined using size-exclusion chromatography and dynamic light scattering. Insertion of fluorine atoms in the polymer side group resulted in an improved solubility in acidic solutions and faster hydrolytic degradation rate, while the ability to self-assemble with an antigenic protein, lysozyme—an important feature of polyphosphazene vaccine adjuvants—was preserved. In vivo assessment of PCPP-F demonstrated its greater ability to induce antibody responses to Hepatitis C virus antigen when compared to its non-fluorinated counterpart. Taken together, the superior immunoadjuvant activity of PCPP-F, along with its improved formulation characteristics, demonstrate advantages of the fluorination approach for the development of this family of macromolecular vaccine adjuvants.

## 1. Introduction

The inclusion of fluorine atoms in drugs and drug candidates remains one of the most indispensable tools for researchers in the field of medicinal chemistry [1,2,3]. Due to its small size and strong electron-withdrawing property, fluorine is widely used to effectively modulate biologically relevant properties of pharmaceuticals. Fluorination can enhance potency and specificity of a drug, modify its metabolic pathways and moderate the dissociation constants of proximal functionalities, therefore creating molecular analogs—bioisosteres, which offer significant advantages [1,2]. The field, which is driven by a deeper understanding of this remarkable element and its ability to improve the pharmacokinetic and pharmacodynamics profiles of drugs, continues to grow. In 2021 only, nine out of fifty new drugs approved by the FDA featured fluorine-containing groups, illustrating the importance of the approach [4].

The insertion of fluorine motifs in macromolecular agents also proves beneficial for drug and vaccine delivery applications, such as gene transfection [5,6]. Fluoropolymers display a superior ability to penetrate cell membranes due to the extremely low free energy of water-interface transfer and limited miscibility with phospholipids, which minimizes their retention within the cell membrane [5,7]. Recent reports also suggest the advantageous effects of omniphobic fluoropolymers for the efficient cytosolic delivery of proteins [7]. Moreover, fluoropolymer-based nanoparticles show an enhanced ability to promote vaccine antigen transportation into the cytosol of dendritic cells, leading to effective antigen cross-presentation [8]. Therefore, fluoropolymers may offer potential advantages for the development of vaccines, for which cross-membrane delivery, dendritic cell activation and antigen presentation constitute essential features of their mechanism of action.

Induction of robust immune responses by subunit vaccines has become increasingly reliant on the aid of immunoadjuvants [9,10,11,12,13]. Water-soluble ionic polyphosphazenes—hybrid organic–inorganic macromolecules—are potent vaccine adjuvants which realize in vivo activity via synergistic antigen delivery and immunostimulation mechanisms [14,15]. Poly[di(carboxylatophenoxy)phosphazene] (PCPP) is an important representative of this class of immunoadjuvants, which has demonstrated its safety and potency in clinical trials [16,17,18,19] and continues to show significant potential with newly emerging antigens or with alternative administration routes, such as microneedle-based intradermal vaccination [20,21,22,23,24,25]. The ability of PCPP to promote immune responses of enhanced magnitude, breadth and durability is closely associated with its exceptional capability to spontaneously self-assemble with antigenic proteins under physiological conditions [21,25,26,27,28]. However, mechanistic aspects of its immunoadjuvant activity are yet to be thoroughly investigated. To that end, synthesis of structural analogs of PCPP presents an important strategy not only for the potential improvement of its in vivo and formulation performance, but also in terms of studying the structure–activity relationship in the system and enhancing our knowledge of the mechanistic aspects of its action as an immunoadjuvant.

The present study reports on the synthesis and physico-chemical characterization of a fluorinated derivative—the bioisostere of the PCPP immunoadjuvant. The results demonstrate that fluorination of polymer side groups resulted in improved formulation characteristics of the macromolecular system without affecting its main biophysical features, such as self-assembly with antigenic protein and ability to undergo hydrolytic degradation at near physiological conditions. In vivo studies with Hepatitis C virus (HCV) antigen revealed the potential of the fluorinated immunoadjuvant in boosting both the magnitude and the quality of the immune response. The results create a basis for further exploration of the fluorinated bioisosteric modification strategy for this class of macromolecular adjuvants.

## 2. Results

### 2.1. Synthesis and Characterization of PCPP-F

**Figure 1:**
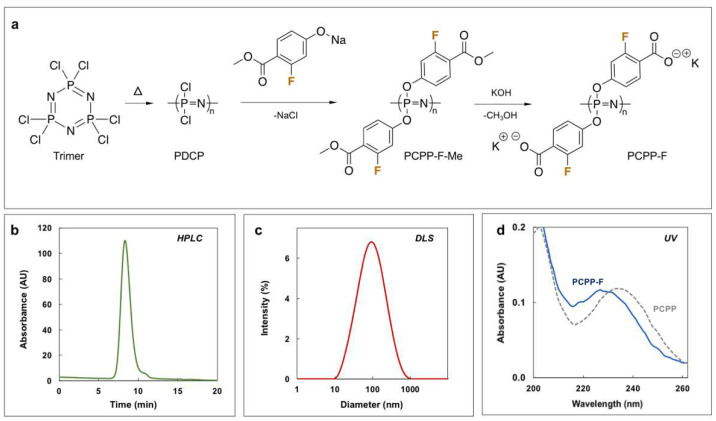
Synthesis and characterization of PCPP-F. (**a**) Synthetic scheme for PCPP-F synthesis, (**b**) SEC-HPLC, (**c**) DLS and (**d**) UV-profiles of PCPP-F (DLS: 0.5 mg/mL; UV: 0.05 mg/mL PCPP-F; 100 mM phosphate buffer, pH 7.4; typical UV spectrum of PCPP is shown as a dashed line for comparison purposes).

PCPP-F was synthesized via a three-step procedure involving ring-opening polymerization of hexachlorocyclotriphosphazene, macromolecular substitution of the resulting polydichlorophosphazene (PDCP) with methyl-2-fluoro-4-hydroxy-benzoate and base-catalyzed hydrolysis of ester functions to yield acid functionalities in the side group (Figure 1a). Although the synthetic route was similar to the one previously described for PCPP [29,30], more mild substitution conditions (50–60 °C vs. 150–160 °C for PCPP) were used in an effort to avoid potential degradation of the polymer. Addition of aqueous potassium hydroxide to the substituted polymer resulted in the formation of precipitate, which was dissolved in water and purified via repeated precipitations in ethanol.

PCPP-F structure and complete substitution/deprotection of the polymer were confirmed using ^1^H, ^31^P and ^19^F NMR spectroscopy (Supplementary Materials, Figure S1). Size-exclusion chromatography (SEC) and dynamic light scattering (DLS) analysis revealed the high molar mass of the polymer and its unimodal distribution (Figure 1b,c and Table 1). The UV spectrum of PCPP-F is practically identical to that of PCPP, although a slight blue shift may be observed for the fluorinated derivative (Figure 1d). This may be indicative of a higher ionization degree of PCPP-F at neutral pH polymer, as hypsochromic shift is typically observed upon dissociation of hydroxybenzoic acid [31].

### 2.2. Aqueous Solubility of PCPP-F

**Figure 2:**
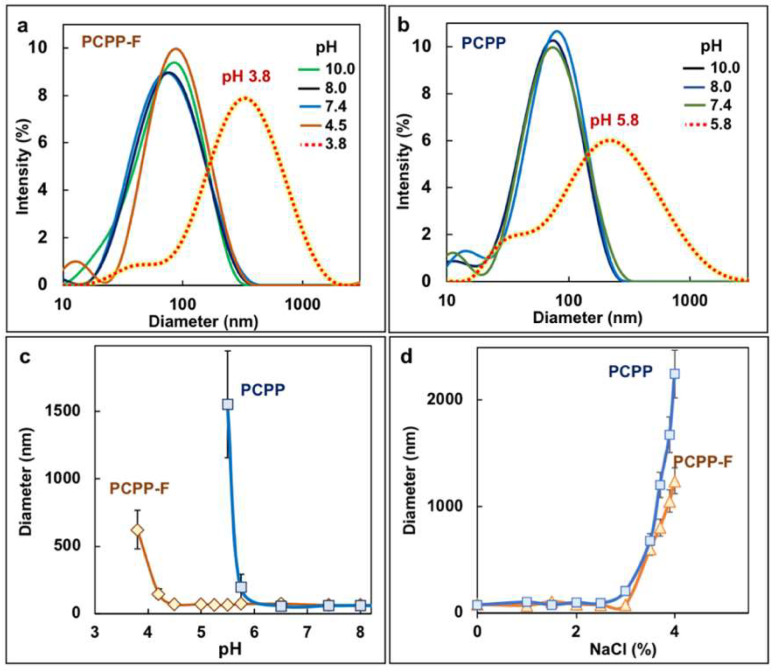
Solubility of PCPP-F. DLS profiles of (**a**) PCPP-F and (**b**) PCPP in aqueous solutions of different pHs and z-average hydrodynamic diameter of PCPP and PCPP-F (**c**) vs. pH of solution (0.5 mg/mL polymer; 100 mM phosphate buffer) and (**d**) vs. concentration of sodium chloride (0.05 mg/mL polymer, pH 7.4).

The solubility of PCPP is generally limited to neutral and basic solutions, which imposes some restrictions on its use with slightly acidic vaccine formulations. Therefore, it was important to evaluate the ability of PCPP-F to resist pH-induced aggregation. Molecular size distributions of PCPP-F and PCPP in solutions of various pHs were compared using the DLS method. Similarly to PCPP, the fluorinated polymer displayed excellent solubility in basic solutions, maintaining its monomodal size distribution (Figure 2a,b). However, the pH response of polymers under acidic conditions revealed substantial differences. While the threshold of aggregation of PCPP was at pH 5.8, PCPP-F showed much stronger resilience, and its phase separation was only observed under significantly more acidic conditions—pH 3.8 (Figure 2a–c). No difference in salt-induced aggregation of polymers was detected (Figure 2d). 

### 2.3. Protein-Binding Capability

**Figure 3:**
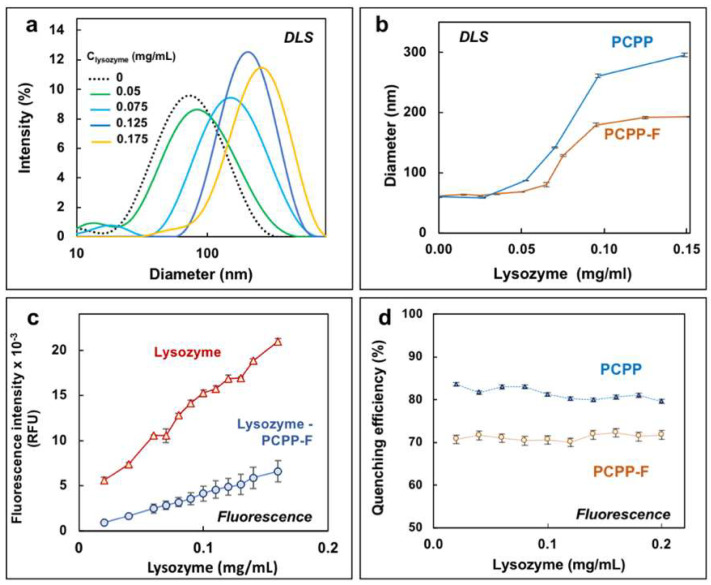
Interactions of PCPP-F with lysozyme. (**a**) DLS profiles of lysozyme–PCPP-F mixtures at different protein concentrations and (**b**) z-average hydrodynamic diameters of lysozyme–PCPP-F and lysozyme–PCPP complexes as a function of protein concentration (0.5 mg/mL polymer, PBS, pH 7.4); (**c**) effect of PCPP-F on the fluorescence of lysozyme (0.05 mg/mL PCPP-F, λ_ex_—300 nm, 100 mM phosphate buffer, pH 7.4); (**d**) comparison of quenching efficiency of PCPP and PCPP-F on lysozyme fluorescence (0.2 mg/mL polymer, 100 mM phosphate buffer, pH 7.4).

The immunoadjuvant potency of PCPP is closely associated with its ability to spontaneously self-assemble with vaccine antigens in aqueous solutions [21,25,26]. Protein-binding characteristics of PCPP-F were evaluated using a model antigenic protein—hen egg lysozyme, which has been intensively studied for its interactions with PCPP [28,32]. Non-covalent association of lysozyme with PCPP is typically manifested in a rise of DLS-detected hydrodynamic dimensions of the complex, with an increase in protein load above a certain threshold. The same general trend was observed for PCPP-F (Figure 3a). Although the dependence of the z-average hydrodynamic diameter of the PCPP-F complex with lysozyme on protein concentration was very similar to that of PCPP, the onset of aggregation was somewhat higher (Figure 3b).

Further analysis of lysozyme binding was conducted using fluorescence spectroscopy by comparing the ability of PCPP and PCPP-F to quench the intrinsic fluorescence of lysozyme. Both polymers were able to significantly decrease the fluorescence intensity of the protein; however, the fluorinated derivative was somewhat less effective than PCPP (Figure 3c and Supplementary Materials, Figure S2). Quenching efficiencies of PCPP and PCPP-F, which were defined as ratios of complex and protein intensities multiplied by 100 and subtracted from 100, are shown in Figure 3d.

### 2.4. Hydrolytic Degradation of PCPP-F

Hydrolytic degradation of polyphosphazene immunoadjuvants is an important prerequisite for their use as injectables [14,33]. The breakdown of the PCPP-F macromolecule in aqueous solution was studied by monitoring its molar mass using SEC and its molecular dimensions using DLS, and the results were compared to the degradation profile of its non-fluorinated analog—PCPP. As the kinetics of PCPP degradation at physiological temperature is relatively slow [29,33], the experiment was conducted under accelerated conditions at 80 °C.

**Figure 4:**
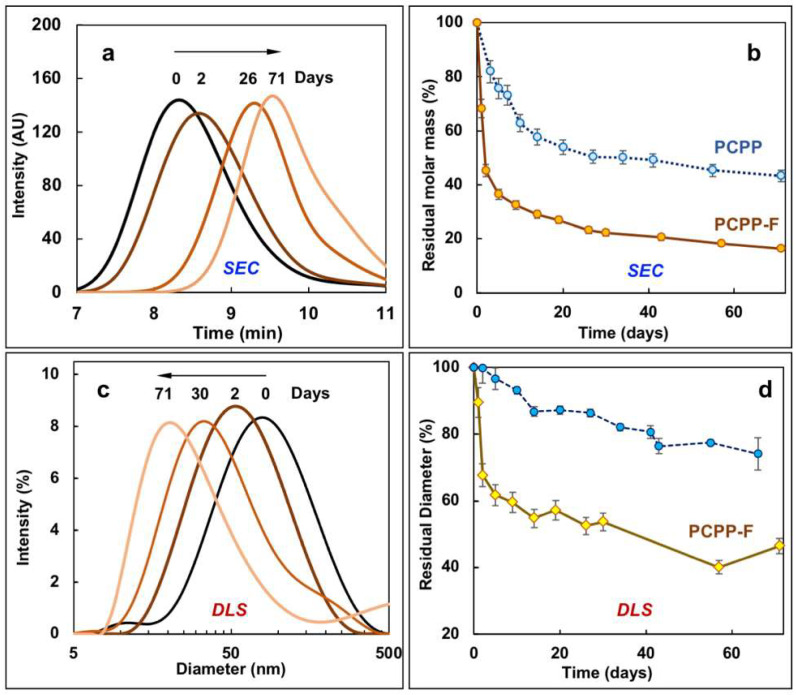
Hydrolytic degradation of PCPP-F. (**a**,**b**) SEC results: (**a**) SEC chromatograms of PCPP-F after polymer incubation in aqueous solution for various time periods and (**b**) residual molar masses of PCPP-F and PCPP as a function of degradation time; (**c**,**d**) DLS data: (**c**) DLS profiles of PCPP-F after polymer incubation in aqueous solution for various time periods and (**d**) residual z-average hydrodynamic diameter of PCPP-F and PCPP as a function of degradation time (residual molar mass and residual diameter are expressed as percentages of the corresponding value at the beginning of the degradation study; 0.25 mg/mL polymer; 80 °C; PBS, pH 7.4).

As seen from Figure 4, SEC and DLS methods show similar degradation trends for PCPP-F. Fifty percent and twenty-five percent decreases in either molar mass or molecular diameter were observed at two and twenty days, correspondingly. The unimodal distribution of molar masses and molecular dimensions was maintained during the degradation process, which is consistent with the data previously reported for PCPP [29,33] However, the kinetics of PCPP-F degradation was notably faster than that observed for non-fluorinated PCPP (Figure 4b). The degradation of both polymers was accompanied with the release of hydroxybenzoic acid (HBA), 2-fluoro-4-hydroxybenzoic acid (F-HBA) (Figure S3) or their low-molecular-weight derivatives, as detected using SEC. As seen from the figures, the faster molar mass loss of PCPP-F in comparison with PCPP was accompanied by a greater release of the side group.

### 2.5. Hemocompatibility of PCPP-F

**Figure 5:**
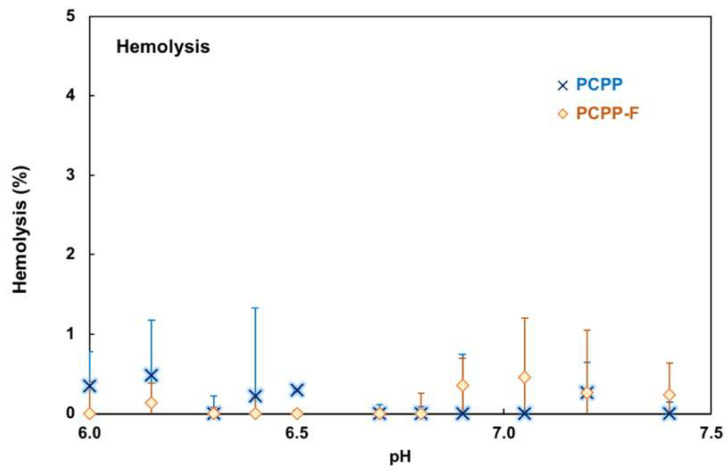
Hemolytic activity of PCPP and PCPP-F in solutions of various pHs (RBCs, PBS, 0.025 mg/mL polymer; pH 7.4, 1 h, 37 °C; errors represent standard deviation, n = 3).

Prior to conducting animal experiments, the hemocompatibility of PCPP-F was evaluated using porcine red blood cells [34,35]. As with PCPP, no hemolytic activity was detected for the fluorinated derivative in the 6.0–7.5 pH range, which suggests a negligible effect of fluorine on polymer hemocompatibility (Figure 5).

### 2.6. In Vivo Activity of PCPP-F

In vivo activity of PCPP-F was evaluated in mice in formulations with soluble Hepatitis C virus (HCV) E2 antigen. The immunoadjuvant potency of PCPP-F was compared with that of non-fluorinated PCPP. The immunization schedule included a prime and three boosts.

**Figure 6:**
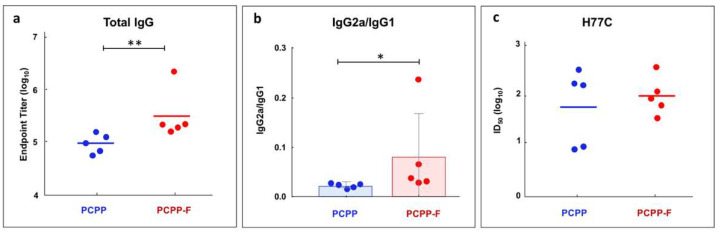
In vivo potency of PCPP-F. Antibody responses induced by PCPP-F and PCPP adjuvanted HCV E2 formulations in vivo: (**a**) serum IgG, (**b**) IgG2a/IgG1 ratio and (**c**) ID_50_ values against homologous HCV isolate (H77) (BALB/c mice, day 28 post immunization, individual values and the geometric mean for the distribution). P values for between-group endpoint titer values were calculated using Kruskal–Wallis analysis of variance with Dunn’s multiple-comparison test, and significant *p* values for comparisons of results between the immunization groups are shown (*, *p* ≤ 0.05; **, *p* ≤ 0.01).

As seen from Figure 6a, IgG titers induced by PCPP-F formulated E2 were statistically significantly higher than those induced by PCPP adjuvanted formulations. An important objective in the development of immunoadjuvants is achieving cell-mediated (T helper 1, Th1) immunity. To that end, IgG subclasses—IgG2a and IgG1 isotypes, which are commonly used as surrogate markers of Th1 and Th2 responses in mice [36,37,38]—were also monitored. Figure 6b shows that PCPP-F formulations were more effective than those of PCPP in shifting the isotype ratio towards a desirable Th1 response. Serum neutralization titers against the homologous HCV isolate (H77) show that PCPP-F formulations were characterized by a tighter clustering of neutralizing responses with less variation within the group of mice (Figure 6c). Elicitation of a more uniform and potent response to the formulated E2 antigen may be a key attribute of the PCPP-F analog.

## 3. Discussion

Synthesis of fluorinated molecules is one of the most common approaches in medicinal chemistry, having proved successful in boosting potency and modulating properties of small drugs [1,2,4]. Fluorination has also been reported to be advantageous in increasing the efficacy of intracellular delivery vehicles [5,7,8]. To that end, introduction of omniphobic, electron-withdrawing fluorine atoms in a vaccine adjuvant presented interest for both potentially boosting its potency and establishing a structure–activity relationship in this class of macromolecules. Copolymers of PCPP containing trifluoroethoxy side groups have been synthesized previously and displayed the appropriate water solubility [39]. However, the random distribution of hydrophobic and hydrophilic segments, along with the established hydrolytic stability of poly[bis(trifluoroethoxy)phosphazene], does not constitute an adequate basis for their use as biodegradable vaccine adjuvants and the establishment of a structure–activity relationship in the system. In contrast, PCPP-F—a water-soluble fluorinated bioisostere of PCPP adjuvant—is a homopolymer which contains two fluorine atoms in each repeat unit. In the present study, the selection of the fluorine atom’s position on the aromatic ring (Figure 1a) was mainly dictated by an intent to achieve its maximal exposure to the antigenic protein in the self-assembly process and to minimize potential complications in the macromolecular substitution step of the polymer synthesis. The polymer was successfully synthesized using the modified synthetic process developed for PCPP and displayed several distinct features differentiating it from the “parent” polymer.

Somewhat counterintuitively, the incorporation of the fluorine atom in the polymer side group, which is expected to enhance omniphobic characteristics of macromolecules, leads to a better polymer stability against aggregation induced by the acidic environment. It was suggested that the inferior solubility of PCPP may result from the unique spatial arrangements of neighboring side groups in the polymer structure [29], which are stabilized by aromatic–aromatic (π–π) association of benzene rings [40,41]. Therefore, it may be hypothesized that the incorporation of fluorine atoms in aromatic rings disturbs such attractions between aromatic systems and reduces their desolvation effect. Regardless of the mechanism, the observed improved solubility of PCPP-F in acidic solutions may prove beneficial for its applications as immunoadjuvant. Typically, it is desirable that the pH of vaccine formulations be kept close to the isoelectric point of the antigen, and therefore, a slightly acidic environment can boost the stability of some vaccines [42,43]. Furthermore, the ability of the immunoadjuvant to withstand microenvironmental pH changes may also be important when there is a need to create combination adjuvants [43]. 

The key feature of polyphosphazene vaccine delivery systems is their ability to effectively bind an antigenic protein payload [14]. This property of PCPP-F was assessed by studying its interactions with lysozyme using DLS and fluorescence spectroscopy. Lysozyme is a model, well-characterized protein which is frequently used as a model vaccine antigen [44,45,46]. Supramolecular dimensions of lysozyme–PCPP-F complexes monitored using DLS over a range of protein concentrations revealed similar profiles for fluorinated and non-fluorinated macromolecules, indicating comparable avidity of both polymers to lysozyme. The insight on the potential differences between PCPP and PCPP-F was further gained using the quenching effect of two polymers on protein fluorescence. The intrinsic fluorescence of lysozyme, which is mainly derived from its tryptophan residues [47], has been a convenient tool for studying the mechanism of interactions with both polymers and small molecules [48,49,50,51]. For example, dextran was reported to quench lysozyme fluorescence by modulating the local environment of tryptophan without affecting the global structure of the protein [51]. Figure 3d and Figure S2 show that although both polymers are effective in decreasing the fluorescence of lysozyme, the quenching efficiency of PCPP-F is lower than that of its non-fluorinated analog. Taken together with data on a somewhat higher threshold of complex aggregation detected using DLS (Figure 3b), it is reasonable to suggest that although the avidities of both polymers to lysozyme are comparable, there could be some distinctions in the conformational behavior of complexes, resulting in a lower proximity of PCPP-F side groups to tryptophan residues which needs to be explored further.

Another important feature of any macromolecular drug which is intended as a component of an injectable pharmaceutical formulation is its ability to undergo hydrolytic degradation. The ability of PCPP-F to undergo molecular breakdown was independently verified using SEC and DLS methods (Figure 4). Both methods showed similar kinetics of the hydrolysis and maintenance of a unimodal distribution of molar mass and molecular diameter. However, the rate of hydrolytical breakdown of PCPP-F was significantly higher than that observed for the breakdown of PCPP under the same conditions. It has been previously established that the mechanism of degradation for PCPP, with its mixed substituent copolymers and structural analogs, includes the release of side groups [29,33,52,53]. Molecular breakdown of PCPP-F was also accompanied by the release of the pendant group—F-HBA. Both molar mass loss (Figure 4b) and the release of side groups (Supplementary Materials, Figure S3) were faster for PCPP-F than for the non-fluorinated analog. The greater hydrolytic instability of PCPP may be discussed in the framework of the established concept of “good leaving group” in the hydrolysis of phosphate esters—a “good” or electron-withdrawing group can be more easily displaced to generate an anion in water [54]. The strong electron-withdrawing effect of the fluorine atom in substituted phenols is well documented and results in a higher dissociation rate of fluorinated derivatives—the pKas of m-fluorophenol and phenol are 9.33 and 9.88, correspondingly [55]. Taken together, the lower pKa of the fluorinated side group (F-HBA is a “better leaving group”) and the faster degradation rate of PCPP-F are consistent with the suggested mechanism of polyphosphazene degradation, which critically depends on the hydrolytic cleavage of the side group for the initiation of the macromolecular breakdown process [33]. The release of the side group in polyphosphazenes is then followed by the generation of the macromolecular hydroxyl derivative, the formation of the unstable phosphazane structure and ultimately the degradation of the polymer chain [33,56].

Immunoadjuvant activity of PCPP-F was evaluated using HCV E2 antigen and was compared to that of PCPP. The results show that the fluorinated macromolecule was significantly more potent in the induction of total IgG titers than its non-fluorinated counterpart (Figure 6a). Directed modulation of the immune response is yet another important objective in the development of immunoadjuvants. Effective vaccines induce both humoral and T cell responses to engender protective antibody and cellular immunity [13]. To that end, achieving T helper 1 (Th1) mediated immunity is highly desirable. Since IgG2a and IgG1 isotypes are usually employed as surrogate markers of Th1 and Th2 responses in mice, respectively [36,37,38], these subclasses were also monitored. Figure 6b shows that PCPP-F was able to shift the response toward a desirable balanced Th1/Th2 immunity, as evaluated using antibody isotype ratio (IgG2a/IgG1).

Taken together, the in vivo and in vitro evaluation of the effect of fluorination on the polyphosphazene adjuvant demonstrate substantial improvements in both the activity and formulation aspects. It needs to be emphasized that the first synthesized fluoro-derivative contained only two fluorine atoms per repeat unit of the polymer. It can be expected that further advancement of this approach can lead to even more profound alterations in biologically relevant properties of the adjuvant. Another potential advantage of fluorination may lie in the application of advanced imaging techniques to explore the mechanistic aspects and metabolic pathways of these adjuvants. Fluorinated molecules are widely used as MRI-detectable tracers for advanced in vivo visualization or for monitoring cellular processes [57,58,59]. The radiolabel tracer atom ^18^F has also been established as a useful positron-emitting isotope in the exquisitely sensitive Positron Emission Tomography (PET) in vivo imaging [2,3].

Although the potential of the approach appears to be substantial, further synthetic efforts need to be conscious of the drawbacks of extensive fluorination, such as decreased polymer solubility or introduction of protein-repellent properties. Therefore, the appropriate fluorination patterns should be identified in order to find the desirable balance between potential advantages of fluorinated polyphosphazene adjuvants and maintenance of their unique features—the ability to self-assemble with vaccine antigens and aqueous formulation homogeneity and stability.

## 4. Materials and Methods

### 4.1. Materials

Propyl paraben, sodium hydride, n-heptane, diglyme, ethanol, sodium phosphate monobasic dihydrate, lysozyme from chicken egg white (Sigma-Aldrich, Saint Louis, MO, USA), phosphate buffered saline, PBS (Thermo Fisher Scientific, Waltham, MA, USA) and hexachlorocyclotriphosphazene (Fushimi Pharmaceutical Co. Ltd., Kagawa, Japan) were used as received. Polydichlorophosphazene (PDCP) was synthesized using a ring-opening polymerization reaction, as described previously [30]. Methyl-2-fluoro-4-hydroxy-benzoate (Accela ChemBio Inc., San Diego, CA, USA) was dried under a vacuum for five days. 

### 4.2. Synthesis of Poly[di(4-carboxylato-3-fluorophenoxy)phosphazene)], PCPP-F

An anhydrous methyl-2-fluoro-4-hydroxy-benzoate (11 g, 64.66 mmol) was dissolved in 50 mL of anhydrous diglyme. A suspension of sodium hydride (1.47 g, 61.42 mmol) in 10 mL of diglyme was slowly added to the methyl-2-fluoro-4-hydroxy-benzoate solution. The resulting sodium salt was added dropwise to PDCP (0.25 g, 4.32 mEq of chlorine atoms) in 20 mL of diglyme and placed in a 500 mL three-neck round-bottom flask equipped with a magnetic stirrer, after which the reaction mixture was kept overnight under nitrogen upon stirring at ambient temperature. The next day, the temperature was increased to 50–60 °C and the reaction was allowed to continue under nitrogen with stirring for 48 h. Aqueous potassium hydroxide (5 mL, 13 M) was added to the reaction mixture, stirred for 1 h and kept overnight at room temperature. The resulting precipitate was collected using centrifugation, redissolved in deionized water (10 mL) and purified repeatedly via precipitating in ethanol (200 mL). The resulting polymer was air-dried at ambient temperature. The yield was 0.625 g (75.3%). The structure of PCPP-F was confirmed using ^1^H NMR, with peak assignments shown below. The single ^31^P NMR peak demonstrates the absence of any residual side groups in the polymer.

^1^H NMR (400 MHz, D₂O): δ [ppm] 6.1 ppm (br, 1H Ar-H), 6.2 ppm (br, 1H Ar-H), 7.1 (br, 1H, Ar-H). 

^31^P NMR: δ [ppm]—19.7 ppm. 

^19^F NMR: δ [ppm]—111.5 ppm. 

### 4.3. Physico-Chemical Characterization

^1^H NMR (400 MHz), ^31^P NMR (161.92 MHz) and ^19^F NMR (376.03 MHz) were recorded using an Ascend Bruker NMR spectrometer (Bruker Biospin Corp, Billerica, MA, USA). Dynamic light scattering (DLS) measurements were conducted using a Malvern Zetasizer Nano series (Malvern Panalytical, Malvern, UK) and data analysis was performed using Malvern Zetasizer 7.10 software (Malvern Instruments Ltd., Worcestershire, UK). Fluorescence spectra were recorded using a BioTek synergy neo2 multi-mode reader (BioTek Instruments, Inc., Winooski, VT, USA). UV-*vis* spectral analysis was conducted using a NanoDrop2000 spectrophotometer (Thermo Scientific, Wilmington, DE, USA). Size-exclusion chromatography (SEC) analysis of molecular masses and polymer degradation studies were conducted using an Agilent 1260 Infinity II Binary LC system equipped with a G7112B binary pump, G7167A Multisampler, G7116A multicolumn thermostat, G7117C diode array detector, G7121A fluorescence detector (Agilent Technologies, Inc., Santa Clara, CA, USA) and TSKgel GMPW size-exclusion column (Tosoh Bioscience, LLC, King of Prussia, PA, USA); PBS with 10% acetonitrile was used as a mobile phase.

### 4.4. Hydrolytic Degradation Studies

Degradation studies were carried out using 0.25 mg/mL solutions of PCPP and PCPP-F in PBS (pH 7.4) at 80 °C. Each formulation was dosed in a 10 mL glass vial, sealed and placed in the Thermolyne Dri-Bath Incubator (Thermo Fisher Scientific, Waltham, MA, USA). Aliquots (100 μL) were taken periodically and analyzed via DLS and SEC-HPLCs. The calibration curve was obtained using narrow poly(acrylic acid) standards ranging from 900 Da to 1500 kDa (American Polymer Standards Corporation, Mentor, OH, USA).

### 4.5. Hemolysis Assay

The hemocompatibility of PCMP was evaluated using a hemolysis test with porcine red blood cells (RBCs, Rockland Immunochemicals, Inc., Limerick, PA, USA) [34,35].

### 4.6. Animal Vaccination

Two groups of five female BALB/c mice (Charles River, Wilmington, MA, USA), age 6 to 8 weeks, were immunized intraperitoneally with 50 μg sE2 formulated with 50 μg of either PCPP-F or PCPP (day 1). Animals then received three booster vaccinations with 10 μg sE2 formulated with 50 μg of either PCPP-F or PCPP on days 7, 14 and 21. Blood samples were collected prior to vaccination on day 1 (pre-bleed) and day 28, processed for serum by centrifugation and stored at −80 °C until analysis was performed.

The study was conducted according to the guidelines of the Declaration of Helsinki and approved by the Institutional Animal Care and Use Committee (IACUC) of Noble Life Sciences Inc., a Maryland, USA-based contract research organization (protocol number: NLS546; approval date: 15 August 2022).

### 4.7. Serological Antibody Detection

To detect sE2-specific total IgG, IgG1 and IgG2a responses in mouse serum, 96-well plates (MaxiSorp, Thermo Fisher, Waltham, MA) were coated overnight with 5 μg/mL Galanthus Nivalis Lectin (Vector Laboratories, Burlingame, CA, USA) at 4 °C. Plates were washed (PBS + 0.05% Tween 20) and coated with 200 ng/well E2 for 2 h at room temperature. After blocking with Pierce™ Protein-Free Blocking Buffer (Thermo Fisher, Waltham, MA, USA) for 1 h, serially diluted sera from mice were added to the plate and incubated for 2 h. The binding of sE2-specific antibodies was detected with 1:5000 dilutions of HRP-conjugated goat anti-mouse secondary antibodies to detect total IgG (H+L), IgG1 and IgG2a (Southern Biotech, Birmingham, AL, USA) with TMB substrate (Bio-Rad Laboratories, Hercules, CA, USA). Absorbance values at 450 nm (SpectraMax M3 microplate reader) were used to determine endpoint titers, which were calculated using curve fitting in GraphPad Prism software and defined as four times the highest absorbance value of pre-immune sera. Significance comparison was performed using a Kruskal–Wallis one-way ANOVA. 

### 4.8. Neutralization Assays

Appropriate dilutions of mouse serum were mixed with HCVpp and incubated at 37 °C for 1 h. The HCVpp–serum mixture was then added to the Huh-7 cells in 96-well plates and incubated at 37 °C for 5 h. After removing the inoculum, the cells were incubated for 72 h with DMEM containing 10% fetal bovine serum (ThermoFisher, Waltham, MA, USA) and the luciferase activity was measured using a Bright-Glo™ assay system (Promega, Madison, WI, USA). Neutralizing antibody (nAb) titers in animal sera were reported as 50% inhibitory dilution (ID_50_) values.

## 5. Conclusions

The advantages of fluorination, which is widely exploited in the field of small-molecule drugs, have yet to be fully explored for vaccine delivery systems. To the best of our knowledge, PCPP-F is the first fluorinated polyphosphazene homopolymer which shows excellent solubility in aqueous solutions and maintains key features of the vaccine delivery vehicle characteristic of clinical stage PCPP. This includes its ability to self-assemble with an antigenic protein and to undergo hydrolytic breakdown in aqueous solutions—an important prerequisite for the development of injectable macromolecules. Most importantly, the insertion of just two fluorine atoms in the repeat group of PCPP results in an improved adjuvant activity in vivo. Further research is needed to establish the boundaries of the aqueous solubility of this class of omniphobic macromolecules—limits within which they can improve biological potency, confirm preclinical safety and elucidate the role fluoropolymers can potentially play in establishing a structure–activity relationship in this class of immunoadjuvants. Furthermore, it can be expected that the presence of adequate levels of fluorine atoms in these macromolecules can advance in vivo visualization using MRI and PET imaging technologies—an invaluable approach for establishing the mechanistic aspects and metabolic pathways of polyphosphazene immunoadjuvants. 

## Figures and Tables

**Table 1:** Molar masses and dimensions of PCPP-F (PBS, pH 7.4).

Molar Mass ^1^			Molecular Dimensions ^2^	
M_w_ (kDa)	M_n_ (kDa)	Đ	D_z_ (nm)	Pdi
750	622	1.2	69	0.41

^1^ as determined by SEC-HPLC, ^2^ as determined by DLS (M_w_—mass-average molar mass; M_n_—number average molar mass; Đ—dispersity; D_z_—z-average hydrodynamic diameter; Pdi—polydispersity index).

## Data Availability

All study data are included in the paper and Supplementary Materials.

## Supplementary Materials

The following supporting information can be downloaded at https://www.mdpi.com/article/10.3390/molecules28104218/s1. Figure S1: NMR spectra of PCPP-F; Figure S2: Fluorescence spectra of (a) lysozyme and (b) lysozyme–PCPP-F formulations in aqueous solutions at various concentrations of protein showing fluorescence quenching effect of PCPP-F; Figure S3: Release of polymer side groups as detected using SEC.

## Sample Availability

Samples of the compounds are not available from the authors.

## References

[1] Johnson B.M., Shu Y.-Z., Zhuo X., Meanwell N.A. (2020). Metabolic and Pharmaceutical Aspects of Fluorinated Compounds. J. Med. Chem..

[2] Gillis E.P., Eastman K.J., Hill M.D., Donnelly D.J., Meanwell N.A. (2015). Applications of Fluorine in Medicinal Chemistry. J. Med. Chem..

[3] Shah P., Westwell A.D. (2007). The role of fluorine in medicinal chemistry. J. Enzyme Inhib. Med. Chem..

[4] He J., Li Z., Dhawan G., Zhang W., Sorochinsky A.E., Butler G., Soloshonok V.A., Han J. (2023). Fluorine-containing drugs approved by the FDA in 2021. Chin. Chem. Lett..

[5] Tan E., Lv J., Hu J., Shen W., Wang H., Cheng Y. (2018). Statistical versus block fluoropolymers in gene delivery. J. Mater. Chem. B.

[6] Wang M., Liu H., Li L., Cheng Y. (2014). A fluorinated dendrimer achieves excellent gene transfection efficacy at extremely low nitrogen to phosphorus ratios. Nat. Commun..

[7] Zhang Z., Shen W., Ling J., Yan Y., Hu J., Cheng Y. (2018). The fluorination effect of fluoroamphiphiles in cytosolic protein delivery. Nat. Commun..

[8] Xu J., Lv J., Zhuang Q., Yang Z., Cao Z., Xu L., Pei P., Wang C., Wu H., Dong Z. (2020). A general strategy towards personalized nanovaccines based on fluoropolymers for post-surgical cancer immunotherapy. Nat. Nanotechnol..

[9] Pulendran B., Arunachalam S.P., O’Hagan D.T. (2021). Emerging concepts in the science of vaccine adjuvants. Nat. Rev. Drug. Discov..

[10] Nanishi E., Dowling D.J., Levy O. (2020). Toward precision adjuvants: Optimizing science and safety. Curr. Opin. Pediatr..

[11] Del Giudice G., Rappuoli R., Didierlaurent A.M. (2018). Correlates of adjuvanticity: A review on adjuvants in licensed vaccines. Semin. Immunol..

[12] McKee A.S., Marrack P. (2017). Old and new adjuvants. Curr. Opin. Immunol..

[13] Reed S.G., Orr M.T., Fox C.B. (2013). Key roles of adjuvants in modern vaccines. Nat. Med..

[14] Andrianov A.K., Langer R. (2021). Polyphosphazene immunoadjuvants: Historical perspective and recent advances. J. Control. Release.

[15] Magiri R., Mutwiri G., Wilson H.L. (2018). Recent advances in experimental polyphosphazene adjuvants and their mechanisms of action. Cell Tissue Res..

[16] Bouveret Le Cam N.N., Ronco J., Francon A., Blondeau C., Fanget B. (1998). Adjuvants for influenza vaccine. Res. Immunol..

[17] Ison M.G., Mills J., Openshaw P., Zambon M., Osterhaus A., Hayden F. (2002). Current research on respiratory viral infections: Fourth International Symposium. Antiviral Res..

[18] Thongcharoen P., Suriyanon V., Paris R.M., Khamboonruang C., de Souza M.S., Ratto-Kim S., Karnasuta C., Polonis V.R., Baglyos L., El Habib R. (2007). A Phase 1/2 Comparative Vaccine Trial of the Safety and Immunogenicity of a CRF01_AE (Subtype E) Candidate Vaccine: ALVAC-HIV (vCP1521) Prime With Oligomeric gp160 (92TH023/LAI-DID) or Bivalent gp120 (CM235/SF2) Boost. J. Acquired Immune Defic. Syndr..

[19] O’Connell R.J., Excler J.-L., Polonis V.R., Ratto-Kim S., Cox J., Jagodzinski L.L., Liu M., Wieczorek L., McNeil J.G., El-Habib R. (2016). Safety and Immunogenicity of a randomized Phase I prime-boost trial with ALVAC-HIV (vCP205) and Oligomeric gp160 MN/LAI-2 Adjuvanted in Alum or Polyphosphazene. J. Infect. Dis..

[20] Romanyuk A., Wang R., Marin A., Janus B.M., Felner E.I., Xia D., Goez-Gazi Y., Alfson K.J., Yunus A.S., Toth E.A. (2023). Skin Vaccination with Ebola Virus Glycoprotein Using a Polyphosphazene-Based Microneedle Patch Protects Mice against Lethal Challenge. J. Funct. Biomater..

[21] Cayatte C., Marin A., Rajani G.M., Schneider-Ohrum K., Snell Bennett A., Marshall J.D., Andrianov A.K. (2017). PCPP-Adjuvanted Respiratory Syncytial Virus (RSV) sF Subunit Vaccine: Self-Assembled Supramolecular Complexes Enable Enhanced Immunogenicity and Protection. Mol. Pharm..

[22] Andrianov A.K., Decollibus D.P., Marin A., Webb A., Griffin Y., Webby R.J. (2011). PCPP-formulated H5N1 influenza vaccine displays improved stability and dose-sparing effect in lethal challenge studies. J. Pharm. Sci..

[23] Andrianov A.K., DeCollibus D.P., Gillis H.A., Kha H.H., Marin A., Prausnitz M.R., Babiuk L.A., Townsend H., Mutwiri G. (2009). Poly[di(carboxylatophenoxy)phosphazene] is a potent adjuvant for intradermal immunization. Proc. Natl. Acad. Sci. USA.

[24] Marin A., Chowdhury A., Valencia S.M., Zacharia A., Kirnbauer R., Roden R.B.S., Pinto L.A., Shoemaker R.H., Marshall J.D., Andrianov A.K. (2021). Next generation polyphosphazene immunoadjuvant: Synthesis, self-assembly and in vivo potency with human papillomavirus VLPs-based vaccine. Nanomedicine.

[25] Andrianov A.K., Marin A., Wang R., Karauzum H., Chowdhury A., Agnihotri P., Yunus A., Mariuzza R.A., Fuerst T.R. (2020). Supramolecular assembly of Toll-like receptor 7/8 agonist into multimeric water-soluble constructs enables superior immune stimulation in vitro and in vivo. ACS Appl. Bio Mater..

[26] Andrianov A.K., Marin A., Fuerst T.R. (2016). Molecular-Level Interactions of Polyphosphazene Immunoadjuvants and Their Potential Role in Antigen Presentation and Cell Stimulation. Biomacromolecules.

[27] Valencia S.M., Zacharia A., Marin A., Matthews R.L., Wu C.-K., Myers B., Sanders C., Difilippantonio S., Kirnbauer R., Roden R.B. (2021). Improvement of RG1-VLP vaccine performance in BALB/c mice by substitution of alhydrogel with the next generation polyphosphazene adjuvant PCEP. Hum. Vaccines Immunother..

[28] Marin A., Taraban M.B., Patel V., Yu Y.B., Andrianov A.K. (2022). Supramolecular Protein-Polyelectrolyte Assembly at Near Physiological Conditions–Water Proton NMR, ITC, and DLS Study. Molecules.

[29] Andrianov A.K., Svirkin Y.Y., LeGolvan M.P. (2004). Synthesis and biologically relevant properties of polyphosphazene polyacids. Biomacromolecules.

[30] Andrianov A.K., Chen J., LeGolvan M.P. (2004). Poly(dichlorophosphazene) as a precursor for biologically active polyphosphazenes: Synthesis, characterization, and stabilization. Macromolecules.

[31] Guo H.-B., He F., Gu B., Liang L., Smith J.C. (2012). Time-Dependent Density Functional Theory Assessment of UV Absorption of Benzoic Acid Derivatives. J. Phys. Chem. A.

[32] Andrianov A.K., Marin A., Deng J., Fuerst T.R. (2020). Protein-loaded soluble and nanoparticulate formulations of ionic polyphosphazenes and their interactions on molecular and cellular levels. Mater. Sci. Eng. C.

[33] DeCollibus D.P., Marin A., Andrianov A.K. (2010). Effect of Environmental Factors on Hydrolytic Degradation of Water-Soluble Polyphosphazene Polyelectrolyte in Aqueous Solutions. Biomacromolecules.

[34] Andrianov A.K., Marin A., Fuerst T.R. (2016). Self-assembly of polyphosphazene immunoadjuvant with poly(ethylene oxide) enables advanced nanoscale delivery modalities and regulated pH-dependent cellular membrane activity. Heliyon.

[35] Yessine M.-A., Lafleur M., Meier C., Petereit H.-U., Leroux J.-C. (2003). Characterization of the membrane-destabilizing properties of different pH-sensitive methacrylic acid copolymers. Biochim. Biophys. Acta Biomembr..

[36] Rostamian M., Sohrabi S., Kavosifard H., Niknam H.M. (2017). Lower levels of IgG1 in comparison with IgG2a are associated with protective immunity against Leishmania tropica infection in BALB/c mice. J. Microbiol. Immunol. Infect..

[37] Grødeland G., Fossum E., Bogen B. (2015). Polarizing T and B Cell Responses by APC-Targeted Subunit Vaccines. Fronti. Immunol..

[38] Visciano M.L., Tagliamonte M., Tornesello M.L., Buonaguro F.M., Buonaguro L. (2012). Effects of adjuvants on IgG subclasses elicited by virus-like Particles. J. Transl. Med..

[39] Andrianov A.K., Marin A., Peterson P., Chen J. (2007). Fluorinated polyphosphazene polyelectrolytes. J. Appl. Polym. Sci..

[40] Martinez C.R., Iverson B.L. (2012). Rethinking the term “pi-stacking”. Chem. Sci..

[41] Grimme S. (2008). Do Special Noncovalent π–π Stacking Interactions Really Exist?. Angew. Chem. Int. Ed. Engl..

[42] Brito L.A., Malyala P., O’Hagan D.T. (2013). Vaccine adjuvant formulations: A pharmaceutical perspective. Semin. Immunol..

[43] Morefield G.L. (2011). A Rational, Systematic Approach for the Development of Vaccine Formulations. AAPS J..

[44] Romero Méndez I.Z., Shi Y., HogenEsch H., Hem S.L. (2007). Potentiation of the immune response to non-adsorbed antigens by aluminum-containing adjuvants. Vaccine.

[45] Temchura V.V., Kozlova D., Sokolova V., Überla K., Epple M. (2014). Targeting and activation of antigen-specific B-cells by calcium phosphate nanoparticles loaded with protein antigen. Biomaterials.

[46] Akiba H., Tamura H., Kiyoshi M., Yanaka S., Sugase K., Caaveiro J.M.M., Tsumoto K. (2019). Structural and thermodynamic basis for the recognition of the substrate-binding cleft on hen egg lysozyme by a single-domain antibody. Sci. Rep..

[47] Imoto T., Forster L.S., Rupley J.A., Tanaka F. (1972). Fluorescence of Lysozyme: Emissions from Tryptophan Residues 62 and 108 and Energy Migration. Proc. Nat. Acad. Sci. USA.

[48] Ding F., Zhao G., Huang J., Sun Y., Zhang L. (2009). Fluorescence spectroscopic investigation of the interaction between chloramphenicol and lysozyme. Eur. J. Med. Chem..

[49] Crouse H.F., Potoma J., Nejrabi F., Snyder D.L., Chohan B.S., Basu S. (2012). Quenching of tryptophan fluorescence in various proteins by a series of small nickel complexes. Dalton Trans..

[50] Revathi R., Rameshkumar A., Sivasudha T. (2016). Spectroscopic investigations on the interactions of AgTiO2 nanoparticles with lysozyme and its influence on the binding of lysozyme with drug molecule. Spectrochim. Acta Part A.

[51] Singh P., Chowdhury P.K. (2017). Unravelling the Intricacy of the Crowded Environment through Tryptophan Quenching in Lysozyme. J. Phys. Chem. B.

[52] Andrianov A.K., Marin A., Peterson P. (2005). Water-soluble biodegradable polyphosphazenes containing N-ethylpyrrolidone groups. Macromolecules.

[53] Andrianov A.K., Marin A., Chen J. (2006). Synthesis, properties, and biological activity of Poly[di(sodium carboxylatoethylphenoxy)phosphazene]. Biomacromolecules.

[54] Kirby A.J., Nome F. (2015). Fundamentals of Phosphate Transfer. Acc. Chem. Res..

[55] Liptak M.D., Gross K.C., Seybold P.G., Feldgus S., Shields G.C. (2002). Absolute pKa Determinations for Substituted Phenols. J. Am. Chem. Soc..

[56] Brito J., Andrianov A.K., Sukhishvili S.A. (2022). Factors Controlling Degradation of Biologically Relevant Synthetic Polymers in Solution and Solid State. ACS Appl. Bio Mater..

[57] Ruiz-Cabello J., Barnett B.P., Bottomley P.A., Bulte J.W.M. (2011). Fluorine (19F) MRS and MRI in biomedicine. NMR Biomed..

[58] Ciliberto M., Maggi F., Treglia G., Padovano F., Calandriello L., Giordano A., Bonomo L. (2013). Comparison between whole-body MRI and Fluorine-18-Fluorodeoxyglucose PET or PET/CT in oncology: A systematic review. Radiol. Oncol..

[59] Jirak D., Galisova A., Kolouchova K., Babuka D., Hruby M. (2019). Fluorine polymer probes for magnetic resonance imaging: Quo vadis?. Magn. Reson. Mater. Phys. Biol. Med..

