# Supplementary material for: Fluorine-Functionalized Polyphosphazene Immunoadjuvant: Synthesis, Solution Behavior and In Vivo Potency
Source: Molecules. 2023 May 21;28(10):4218. doi: 10.3390/molecules28104218 (PMC10221343; doi:10.3390/molecules28104218)
Supplement: Supplementary file 1 [file molecules-28-04218-s001.zip › molecules-2404845-supplementary.pdf]

Supplementary Materials

Fluorine-Functionalized Polyphosphazene Immunoadjuvant: Synthesis, Solution Behavior and *In Vivo* Potency

Harichandra D. Tagad [1], Alexander Marin [1], Ruixue Wang [1,] Abdul S. Yunus [1], Thomas R. Fuerst [1, 2] and Alexander K Andrianov [1,*]

[1] Institute for Bioscience and Biotechnology Research, University of Maryland Rockville, MD 20850, USA
[2] Department of Cell Biology and Molecular Genetics, University of Maryland, College Park, MD 20742, USA
* Correspondence: aandrianov@umd.edu

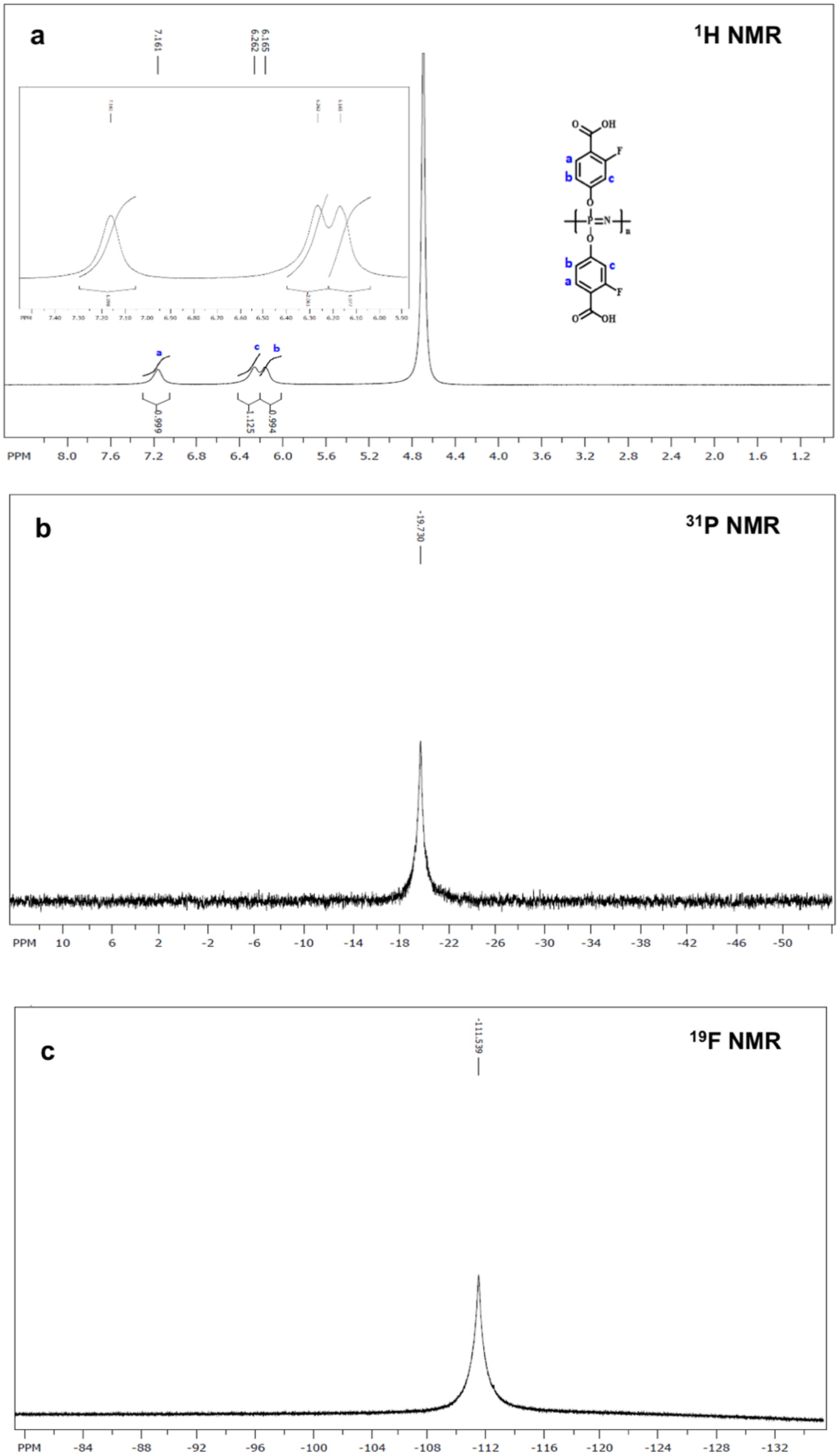

Figure S1. (**a**) ^{1}H, (**b**) ^{31}P and (**c**) ^{19}F NMR spectra of PCPP-F (D_2O).

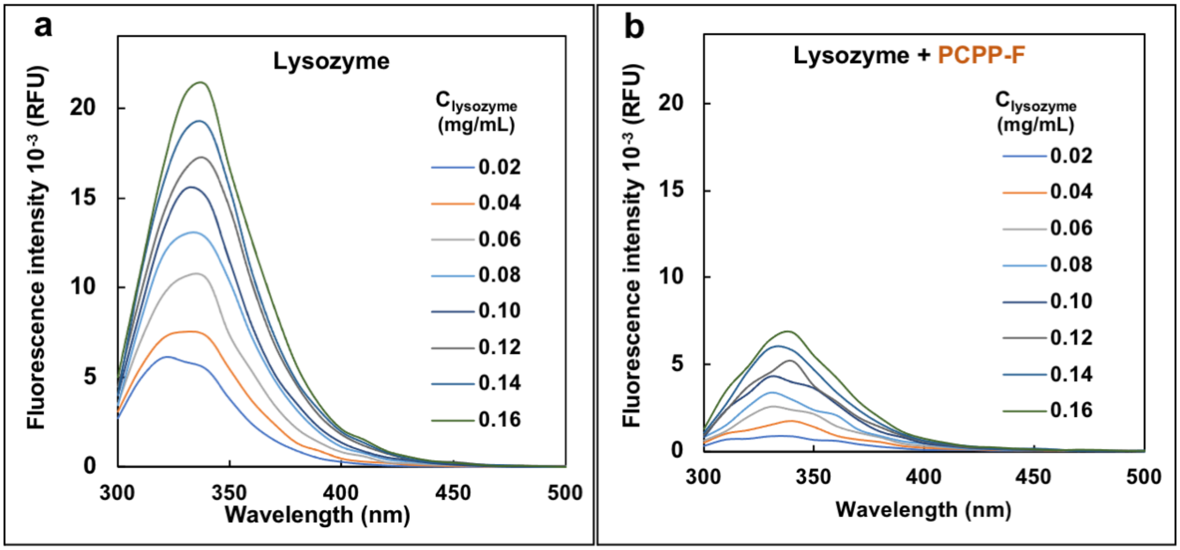

Figure S2. Fluorescence spectra of (**a**) lysozyme and (b) lysozyme - PCPP-F formulations in aqueous solutions at various concentration of protein showing fluorescence quenching effect of PCPP-F (0.05 mg/mL PCPP-F; phosphate buffer, pH 7.4; λ_{ex} - 300 nm).

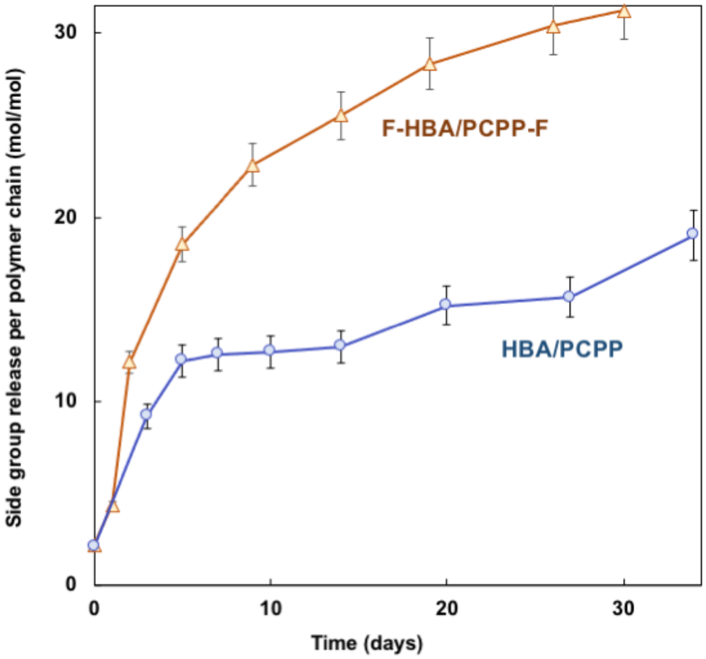

Figure S3. Release of low molecular weight products containing polymer side groups as detected by SEC: hydroxybenzoic acid (HBA) of PCPP and fluorinated hydroxybenzoic acid (F-HBA) of PCPP-F (0.25 mg/mL polymer; 80°C; PBS, pH 7.4).
